# Clinical significance of cytogenetic and molecular genetic abnormalities in 634 Chinese patients with myelodysplastic syndromes

**DOI:** 10.1002/cam4.3786

**Published:** 2021-02-20

**Authors:** Xuefen Yan, Lu Wang, Lingxu Jiang, Yingwan Luo, Peipei Lin, Wenli Yang, Yanling Ren, Liya Ma, Xinping Zhou, Chen Mei, Li Ye, Gaixiang Xu, Weilai Xu, Haiyang Yang, Chenxi Lu, Jie Jin, Hongyan Tong

**Affiliations:** ^1^ Department of Hematology The First Affiliated Hospital Zhejiang University School of Medicine Hangzhou Zhejiang China; ^2^ Myelodysplastic Syndromes Diagnosis and Therapy Center The First Affiliated Hospital Zhejiang University School of Medicine Hangzhou Zhejiang China; ^3^ Department of Hematology People's Hospital of Quzhou Quzhou Zhejiang China; ^4^ Department of Radiotherapy Taizhou Central Hospital (Taizhou University Hospital Taizhou Zhejiang China

**Keywords:** genetic mutations, karyotype, myelodysplastic syndromes, trisomy 8

## Abstract

**Purpose:**

To explore the relevance of cytogenetic or molecular genetic abnormalities to clinical variables, including clinical and laboratory characteristics and prognosis in Chinese patients with myelodysplastic syndromes (MDS).

**Methods:**

A total of 634 consecutive patients diagnosed with MDS at The First Affiliated Hospital, Zhejiang University School of Medicine from June 2008 to May 2018 were retrospectively included in this study. All patients had evaluable cytogenetic analysis, and 425 patients had MDS‐related mutations sequencing.

**Results:**

38.6% of patients displayed abnormal karyotypes. The most common cytogenetic abnormality was +8 (31%). Sole +8 was related to female (*p* = 0.002), hemoglobin >10 g/dL (*p* = 0.03), and <60 years old (*p* = 0.046). *TP53* mutations were associated with complex karyotype (CK) (*p* < 0.001). *DNMT3A* mutations correlated with ‐Y (*p* = 0.01) whereas *NRAS* mutations correlated with 20q‐ (*p* = 0.04). The overall survival (OS) was significantly inferior in patients with +8 compared with those with normal karyotype (NK) (*p* = 0.003). However, the OS of sole +8 and +8 with one additional karyotypic abnormality was not different from NK (*p* = 0.16), but +8 with two or more abnormalities had a significantly shorter OS than +8 and +8 with one additional karyotypic abnormality (*p* = 0.02). In multivariable analysis, ≥60 years old, marrow blasts ≥5% and *TP53* mutations were independent predictors for poor OS (*p* < 0.05), whereas *SF3B1* mutations indicated better prognosis. Male *IDH1* and *IDH2* mutations and marrow blasts ≥5% were independent risk factors for worse leukemia free survival (LFS) (*p* < 0.05).

**Conclusion:**

In this population of Chinese patients, trisomy 8 is the most common karyotypic abnormality. Patients with +8 showed a poorer OS compared with patients with NK. Sole +8 and +8 with one additional karyotypic abnormality had similar OS with NK, whereas +8 with two or more abnormalities had a significantly shorter OS. *DNMT3A* mutations correlated with ‐Y and *NRAS* mutations correlated with 20q‐. *TP53* mutations were associated with CK and had a poor OS. *SF3B1* mutations indicated a favorable OS. *IDH1* and *IDH2* mutations independently indicated inferior LFS.

## Graphical abstract

This study aimed to explore the relevance of cytogenetic or molecular genetic abnormalities to clinical variables, including clinical and laboratory characteristics and prognosis in patients with myelodysplastic syndromes (MDS) and showed that cytogenetic changes had a potential correlation with molecular genetic abnormalities and both had significant influence on the prognosis of MDS.

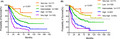

## 1 INTRODUCTION

Myelodysplastic syndromes (MDS) are a heterogeneous group of hematopoietic stem cell malignancies characterized by ineffective hematopoiesis resulting in peripheral cytopenia, and a propensity to evolve into acute myeloid leukemia (AML).^1^, ^2^ About 50–60% of patients exhibit acquired cytogenetic abnormalities.^3^, ^4^, ^5^ 5q‐, +8, −7/7q‐, 20q‐, ‐Y are the most common abnormal karyotypes.^6^, ^7^, ^8^ The occurrence of +8 is the most common cytogenetic abnormality in Chinese patients,^9^, ^10^, ^11^, ^12^, ^13^ which is much higher than that in European and American patients (30–37.8% *vs*. 11.3–16.0%).^6^, ^8^, ^10^ The revised international prognosis scoring system (IPSS‐R) assigned +8 into the intermediate risk group.^14^ However, patients with +8 are prognostically different with median overall survival (OS) from 5.9 to 26 months,^15^, ^16^, ^17^ which is partly depending on the racial background. Median OS of patients with sole +8, varying from 32.5 to 85.9 months, are even harder to predict.^18^, ^19^ Patients with +8 karyotype still need further research explorations.

With the use of next generation sequencing (NGS) technologies, 70–90% of MDS patients were detected with one or more genetic mutations.^20^, ^21^, ^22^, ^23^, ^24^ Mutations were found to be associated with clinical phenotypes and prognosis in MDS patients.^25^ The correlation between aberrant karyotypes and genetic mutations has been described previously. Mutations in *U2AF1*, *ASXL1*, *IDH*, and *ZRSF2* were reported to be clustered with +8,^26^, ^27^, ^28^, ^29^ whereas *SRSF2*, *ASXL1*, and *U2AF1* mutations were associated with 20q‐.^30^ However, the results were from single‐center studies. Therefore, the conclusions needed to be confirmed.

This study aimed to analyze the relationships between aberrant karyotypes and genetic mutations in a cohort of 634 native born Chinese MDS patients, and explore their associations with clinical features and prognosis in MDS patients.

## 2 METHODS

### 2.1 Patients and diagnostic criteria

Six hundred and thirty‐four patients were selected from the institutional database of patients with primary MDS from June 2008 to May 2018. Study eligibility criteria included the availability of bone marrow (BM) smear, BM histology, and cytogenetic information at the time of diagnosis/new referral to the hospital. Even though the patients at first referral were diagnosed at other hospitals, they were re‐examined and not received treatment until hospitalized in our center. Clinical and laboratory data were acquired at the time of diagnosis. The diagnoses of MDS were according to the 2016 WHO classification.^1^ The current study was approved by the ethics committee of The First Affiliated Hospital, Zhejiang University School of Medicine.

### 2.2 Cytogenetic analysis

Cytogenetic analysis was done according to the International System for Human Cytogenetic Nomenclature (ISCN) either 2005 or 2013. A total of 427 (67.4%) patients had grown 20 metaphases. The other 207 (32.6%) patients had grown 3–19 metaphases. Fluorescence in situ hybridization (FISH) for abnormalities of chromosomes 5, 7, 8, 20 was undertaken in 74 patients. The presence of three or more distinct numerical or structural cytogenetic abnormalities was considered as complex karyotype (CK). Chromosomal abnormalities were considered clone if the same structural abnormality and extra chromosome appeared in at least two metaphases. Monosomy was recurrent in at least three metaphases.

### 2.3 Mutation analysis

A total of 425 patients had DNA sequencing to detect recurrent genetic mutations in MDS. Next generation sequencing (NGS) of PCR‐amplified exons of 15 genes, *TP53*, *EZH2*, *SF3B1*, *U2AF1*, *NRAS*, *DNMT3A*, *IDH1*, *IDH2*, *TET2*, *CBL*, *ETV6*, *JAK2*, *SRSF2*, *RUNX1*, and *ASXL1*, was performed in 223 patients. Sanger's method sequencing was performed in 202 patients for detecting six genetic mutations, including *DNMT3A*, *SF3B1*, *SRSF2*, *IDH1*, *IDH2*, and *U2AF1*. Known single‐nucleotide polymorphisms (SNPs), intronic polymorphisms more than six bases from a splice junction, and variable allele frequency (VAF) <2% were excluded from further analysis.

### 2.4 Prognostic criteria, response, and follow up

Patients were assigned into prognostic risk groups according to the IPSS‐R.^14^ The options of treatments included supportive care, low‐intensity treatment approach hypomethylation agents (HMA)±chemotherapy (HMA±chemo), or allogeneic hematopoietic stem cell transplantation (allo‐HSCT) according to NCCN guideline. Response to treatment was defined per the 2006 revised international working group (IWG) response criteria.^31^ OS was measured from the time of diagnosis to the time of death from any cause. LFS was calculated from the date of diagnosis to the date of leukemia transformation.

### 2.5 Statistics

Statistical significance was analyzed using Student's *t*‐test to compare differences of the continuous variables in normal distribution. Mann–Whitney test was used for the comparison of continuous variables in abnormal distribution. Patient groups with nominal variables were compared by chi‐square test or Fisher exact test (less than 5 cases per group). Wilcoxon rank sum test or trend test was used for comparison of contingency table. OS and LFS curves were plotted using the Kaplan–Meier estimation and compared by the log‐rank test. Cox proportional hazard regression model was used to calculate independent factors for OS and LFS in multivariable analysis. All p values were calculated with the use of two‐sided tests and less than 0.05 were considered significant. All calculations were performed using R programming language (version 3.5.1).

## 3 RESULTS

### 3.1 Cytogenetic abnormalities

In total, 634 primary MDS patients with cytogenetic reports were identified, including 369 males and 265 females, of whom the median age was 57 years old. Table 1 summarizes the clinical and laboratory characteristics of all patients stratifying by karyotypes.

**TABLE 1:** Clinical and laboratory characteristics of 634 MDS patients.

	All, N = 634	Normal karyotype, N = 389	Aberrant karyotype, N = 245	p
Gender (%)				
Male	369 (58.2)	217 (55.8)	152 (62.0)	0.12
Female	265 (41.8)	172 (44.2)	93 (38.0)	
Age in years, median (range)	57 (18–86)	55 (18–86)	57 (18–86)	0.08
Absolute neutrophil count ×10^9^/L, median (range)	1.2 (0–25)	1.2 (0–24.4)	1.1 (0.04–25)	0.18
Hemoglobin g/L, median (range)	76 (22–158)	77 (23–158)	75 (22–139)	0.29
Platelet count ×10^9^/L, median (range)	56 (3–1534)	62 (3–976)	47 (4–1534)	0.30
Bone marrow blasts (%)				
Median (range)	5 (0–19)	4 (0–19)	6 (0–19)	<0.001
<5%	328 (51.7)	223 (57.3)	105 (42.9)	
≥5%	306 (48.3)	166 (42.7)	140 (57.1)	
WHO (2016) subtype (%)				
MDS‐SLD	64 (10.1)	52 (13.4)	12 (4.9)	<0.001
MDS‐RS‐SLD	24 (3.8)	20 (5.1)	4 (1.6)	
MDS‐RS‐MLD	13 (2.1)	7 (1.8)	6 (2.4)	
MDS‐MLD	218 (34.4)	144 (37.0)	74 (30.2)	
MDS‐del(5q)	3 (0.5)	0	3 (1.2)	
MDS‐EB‐1	154 (24.3)	87 (22.4)	67 (27.3)	
MDS‐EB‐2	152 (24.0)	79 (20.3)	73 (29.8)	
MDS‐U	6 (0.9)	0	6 (2.4)	
IPSS‐R score (%)				
Very low	17 (2.7)	13 (3.3)	4 (1.6)	<0.001
Low	158 (24.9)	134 (34.4)	24 (9.8)	
Intermediate	166 (26.2)	121 (31.1)	45 (18.4)	
High	162 (25.6)	101 (26.0)	61 (24.9)	
Very high	131 (20.7)	20 (5.1)	111 (45.3)	

Abbreviations: IPSS‐R, Revised International Prognostic Scoring System; MDS‐del(5q), MDS with isolated del(5q); MDS‐EB‐1, MDS with excess blasts‐1; MDS‐EB‐2, MDS with excess blasts‐2; MDS‐MLD, MDS with multilineage dysplasia; MDS‐RS‐MLD, MDS with ring sideroblasts and multilineage dysplasia; MDS‐RS‐SLD, MDS with ring sideroblasts and single‐lineage dysplasia; MDS‐SLD, MDS with single‐lineage dysplasia; MDS‐U, MDS, unclassifiable; WHO, World Health Organizations.

Two hundred and forty‐five patients (38.6%) displayed abnormal karyotypes, including 62 (25.3%) sole numerical abnormalities, 74 (30.2%) sole structural abnormalities, and 109 (44.5%) harboring both. The data showed that the most common abnormality was trisomy 8 (+8) (12.0%), followed by −5/5q‐ (10.6%), monosomy 7 and deletion 7q (−7/7q‐) (7.7%), deletion 20q (20q‐) (6.3%), monosomy 13 and deletion 13q (−13/13q‐) (2.8%), monosomy 11 and deletion 11q (−11/11q‐) (2.7%), monosomy 18 (−18) (2.5%), deletion Y (‐Y) (2.2%), and monosomy 3 (−3) (2.1%). Other cytogenetic abnormalities included trisomy 6 (+6), Y (+Y), 9 (+9), 16 (+16) and monosomy 10 (−10), deletion 12p (12p‐), isochromosome 17q (i [17q]) and so on.

### 3.2 Phenotypic correlates

The karyotype of patients correlated with marrow blasts, WHO‐subtype, and IPSS‐R group (*p* < 0.001, respectively; Table 1). The comparison between CK and non‐CK was performed and given in Table 2. The CK was associated with male gender (*p* < 0.001), ≥60 years old (*p* = 0.01), PLT <50 × 10^9^/L (*p* < 0.001), and IPSS‐R (*p* < 0.001). Seventy‐six patients had trisomy 8, which accounted for 31% in patients with karyotype abnormalities whereas 12% in all patients included in the current study. Among them, 42 (55.3%) had sole +8, and 34 (44.7%) had +8 with additional abnormalities.

**TABLE 2:** Clinical and laboratory characteristics of 245 MDS patients with aberrant karyotype.

	Aberrant karyotype, N = 245	Complex karyotype, N = 95	Non‐complex karyotype, N = 150	*P*
Gender (%)				
Male	152 (62.0)	74 (77.9)	78 (52.0)	<0.001
Female	93 (38.0)	21 (22.1)	72 (48.0)	
Age in years, median (range)	57 (18–86)	62 (18–84)	55 (19–86)	0.01
Absolute neutrophil count ×10^9^/L, median (range)	1.1 (0.04–25)	1.1 (0.04–9)	1.2 (0.1–25)	0.07
Hemoglobin g/L, median (range)	75 (22–139)	70 (40–139)	76 (22–130)	0.10
Platelet count ×10^9^/L, median (range)	47 (4–1534)	36 (5–366)	57 (4–1534)	<0.001
Bone marrow blasts (%)				
Median (range)	6 (0–19)	7 (0–19)	5.5 (0–19)	0.26
<5%	105 (42.9)	36 (37.9)	69 (46.0)	
≥5%	140 (57.1)	59 (62.1)	81 (54.0)	
WHO (2016) subtype (%)				
MDS‐SLD	12 (4.9)	2 (2.1)	10 (6.7)	0.32
MDS‐RS‐SLD	4 (1.6)	0	4 (2.7)	
MDS‐RS‐MLD	6 (2.4)	3 (3.2)	3 (2)	
MDS‐MLD	74 (30.2)	29 (30.5)	45 (30)	
MDS‐del(5q)	3 (1.2)	0	3 (2)	
MDS‐EB‐1	67 (27.3)	29 (30.5)	38 (25.3)	
MDS‐EB‐2	73 (29.8)	29 (30.5)	44 (29.3)	
MDS‐U	6 (2.4)	3 (3.2)	3 (2)	
IPSS‐R score (%)				
Very low	4 (1.6)	0	4 (2.7)	<0.001
Low	24 (9.8)	0	24 (16.0)	
Intermediate	45 (18.4)	6 (6.3)	39 (26.0)	
High	61 (24.9)	12 (12.6)	49 (32.7)	
Very high	111 (45.3)	77 (81.1)	34 (22.7)	

Abbreviations: IPSS‐R, Revised International Prognostic Scoring System; MDS‐del(5q), MDS with isolated del(5q); MDS‐EB‐1, MDS with excess blasts‐1; MDS‐EB‐2, MDS with excess blasts‐2; MDS‐MLD, MDS with multilineage dysplasia; MDS‐RS‐MLD, MDS with ring sideroblasts and multilineage dysplasia; MDS‐RS‐SLD, MDS with ring sideroblasts and single‐lineage dysplasia; MDS‐SLD, MDS with single‐lineage dysplasia; MDS‐U, MDS, unclassifiable; WHO, World Health Organizations.

Sole +8 was associated with <60 years old (*p* = 0.046), female distribution (*p* = 0.002), and hemoglobin >10 g/dL (*p* = 0.03) compared with karyotypic abnormalities without sole +8. Although significant differences exist in five subgroups of IPSS‐R between patients with sole +8 and patients with other karyotypic abnormalities (*p* = 0.005), there was no significant difference between the two groups in the distribution of IPSS‐R subtypes (*p* = 0.63; Table 3).

**TABLE 3:** Clinical and laboratory features between MDS patients with isolated +8 and other abonormalities.

	Isolated +8, N (%)	Abnormal karyotype except isolated +8, N (%)	*P*
	42 (17.1)	203 (82.9)	
Gender, M/F	17/25	135/68	0.002
Age, years			
<60	29 (69.0)	103 (50.7)	0.046
≥60	13 (31.0)	100 (49.3)	
WHO (2016) subtype			
MDS‐SLD	4 (9.5)	8 (3.9)	0.100
MDS‐RS‐SLD	0	4 (2.0)	
MDS‐RS‐MLD	3 (7.1)	3 (1.5)	
MDS‐MLD	13 (31.0)	61 (30.0)	
MDS‐5q‐	0	3 (1.5)	
MDS‐EB‐1	14 (33.3)	53 (26.1)	
MDS‐EB‐2	7 (16.7)	66 (32.5)	
MDS‐U	1 (2.4)	5 (2.5)	
Lineage counts of cytopenia			
Single lineage	13 (31.0)	42 (20.6)	0.305
Two lineages	13 (31.0)	81 (40.0)	
There lineages	16 (38.0)	80 (39.4)	
Hemoglobin, g/L			
<80	26 (61.9)	120 (59.1)	0.030
80–100	3 (7.1)	46 (22.7)	
>100	13 (31.0)	37 (18.2)	
Absolute neutrophil count (×10^9^/L)			
<0.8	17 (40.5)	57 (28.1)	0.269
≥0.8	25 (59.5)	146 (71.9)	
Platelet count (×10^9^/L)			
<50	21 (50.0)	103 (50.7)	0.280
50–100	4 (9.5)	37 (18.2)	
>100	17 (40.5)	63 (31.0)	
Bone marrow blast percentage			
<5%	21 (50.0)	84 (41.4)	0.390
≥5%	21 (50.0)	119 (58.6)	
IPSS‐R score			
Very low	0	4 (2.0)	0.005
Low	3 (7.1)	21 (10.3)	
Intermediate	14 (33.3)	31 (15.3)	
High	15 (35.7)	46 (22.7)	
Very high	10 (23.8)	101 (49.8)	
Risk stratification			
Lower risk	5 (11.9)	33 (16.3)	0.630
Higher risk	37 (88.1)	170 (83.7)	

Abbreviations: F, female; M, male.

We further divided 76 patients with +8 abnormality into three groups: sole +8 group (tri8, 42), +8 with one karyotypic abnormality (tri8^+1^, 11), and +8 with two or more abnormalities (tri8^+≥2^, 23). Patients in tri8^+1^ group did not have abnormalities concerning chromosome 7. Among 42 patients with sole +8, 5 patients were ranked as lower risk (LR) and received supportive care. The other 37 patients, in the higher risk (HR) group received various treatments as follows: supportive treatment,^22^ HMA±chemo,^14^ and allo‐HSCT.^1^ Eleven patients with tri8^+1^ were all in the HR group, of which eight patients received supportive care, two patients received HMA±chemo, and one received allo‐HSCT. Twenty‐three patients with tri8^+≥2^ were all in the HR group. Among them, 17 received supportive treatment, 6 received HMA±chemo.

There was a significant difference in the three groups with respect to age (*p* = 0.005; Table 4). By pairwise comparison of the three groups, it was found that tri8^+1^ was more common in young patients (< 60 years old) than those with tri8 (*p* = 0.005) and tri8^+≥2^ (*p* = 0.003). There were also significant differences in neutrophil count among the three groups (*p* = 0.02). Compared with tri8, patients in tri8^+1^ were associated with neutrophil ≥0.8 × 10^9^/L (*p* = 0.02).

**TABLE 4:** Clinical and laboratory features of MDS patients with trisomy 8.

	Tri8, N (%)	Tri8^+1^, N (%)	Tri8^+≥2^, N (%)	*P*
	42 (55.3)	11 (14.5)	23 (30.3)	
Gender, M/F	17/25	6/5	14/9	0.270
Age (years)				
<60	29 (69.0)	11 (100)	11 (47.8)	0.005
≥60	13 (31.0)	0	12 (52.2)	
WHO (2016) subtype				
MDS‐SLD	4 (9.5)	0	0	0.560
MDS‐RS‐SLD	0 (0)	0	0	
MDS‐RS‐MLD	3 (7.1)	0	1 (4.3)	
MDS‐MLD	13 (31.0)	2 (18.2)	10 (43.5)	
MDS‐5q‐	0	0	0	
MDS‐EB‐1	14 (33.3)	6 (54.5)	5 (21.7)	
MDS‐EB‐2	7 (16.7)	3 (27.3)	6 (26.1)	
MDS‐U	1 (2.4)	0	1 (4.3)	
Lineage counts of cytopenia				
Single lineage	13 (31.0)	4 (36.4)	5 (21.7)	0.470
Two lineages	13 (31.0)	3 (27.3)	4 (17.4)	
Three lineages	16 (38.1)	4 (36.4)	14 (60.9)	
Absolute neutrophil count (×10^9^/L)				
<0.8	17 (40.5)	0	7 (30.4)	0.020
≥0.8	25 (59.5)	11 (100)	16 (69.6)	
Hemoglobin, g/L				
<80	26 (61.9)	6 (54.5)	16 (69.6)	0.720
80–100	3 (7.1)	2 (18.2)	2 (8.7)	
>100	13 (31.0)	3 (27.3)	5 (21.7)	
Platelet count (×10^9^/L)				
<50	21 (50)	5 (45.5)	16 (69.6)	0.110
50–100	4 (9.5)	3 (27.3)	4 (17.4)	
>100	17 (40.5)	3 (27.3)	3 (13.0)	
Bone marrow blast percentage				
<5%	21 (50.0)	2 (18.2)	12 (52.2)	0.130
≥5%	21 (50.0)	9 (81.8)	11 (47.8)	
IPSS‐R Score				
Very low	0	0	0	<0.001
Low	3 (7.1)	0	0	
Intermediate	14 (33.3)	3 (27.3)	1 (4.3)	
High	15 (35.7)	5 (45.5)	4 (17.4)	
Very high	10 (23.8)	3 (27.3)	18 (78.3)	
Risk stratification				
Relatively low	5 (11.9)	3 (27.3)	1 (4.3)	0.130
Relatively high	37 (88.1)	8 (72.7)	22 (95.7)	

### 3.3 Molecular correlates

A total of 425 patients were examined for genetic mutations. Of which 204 (48.0%) patients were identified carrying one or more mutations. The frequencies of mutated genes were *TET2* (14.8%, 33/223), *TP53* (12.6%, 28/223), *SF3B1* (10.8%, 43/399), *RUNX1* (10.8%, 24/223), *U2AF1* (10.7%, 44/410), *ASXL1* (9.4%, 21/223), *JAK2* (5.8%, 13/223), *DNMT3A* (5.1%, 21/408), *EZH2* (4.9%, 11/223), *SRSF2* (4.0%, 17/423), *CBL* (4.0%, 9/223), *NRAS* (3.6%, 8/223), *IDH1* (3.1%, 13/422), *IDH2* (2.6%, 11/422), and *ETV6* (0.9%, 2/223). Forty‐nine out of 76 patients with +8 received DNA sequencing, and 22 (44.9%) were detected carrying ≥1 related genetic mutation.

In the current study, we categorized karyotypes into nine groups: NK, +8, 20q‐/‐20, −5/5q‐, −7/7q‐, ‐y, 11q‐, CK (≥3 abnormalities), and other abnormalities. *DNMT3A* mutations correlated with ‐Y (*p* = 0.01), *NRAS* mutations were related to 20q‐ (*p* = 0.04), and *TP53* mutations were associated with CK (*p* < 0.001). Mutational frequency of *TP53* in IPSS‐R cytogenetic prognostic subsets was 0 in very good, 2 in good (3.0%), 3 in intermediate (6.0%), 7 in poor (11.5%), and 16 in very poor (41.0%), indicating that *TP53* mutational frequency ascended as karyotype risk increased (*p* < 0.001).

### 3.4 Prognostic relevance

**FIGURE 1:**
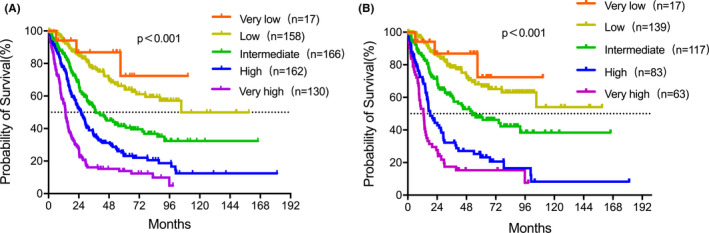
Overall survival of patients according to the IPSS‐R risk category. (A) For all patients, median OS was 32.2 (95% CI: 29.3–39.6) months and decreases as prognostic risk increases (*p* < 0.001). (B) After censoring patients who received treatments (HMA, chemotherapy, and allo‐HSCT), OS was 48.4 (95% CI: 43.6–53.7) months and markedly associated with IPSS‐R (*p* < 0.001).

**FIGURE 2:**
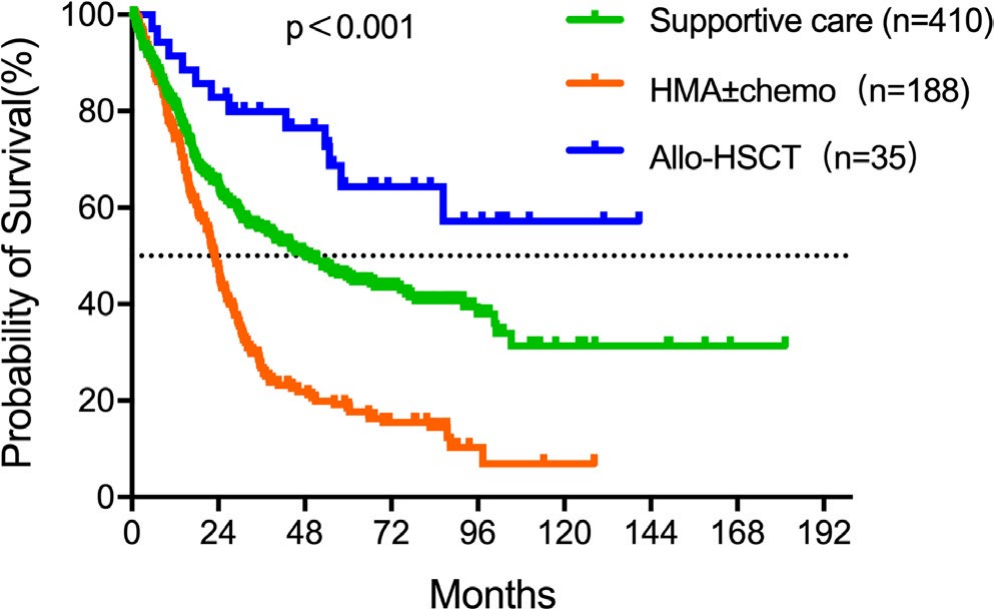
Kaplan–Meier curves for OS of patients stratified by different treatments. Median OS were 48.0 (95% CI: 43.2–53.3) months and 22.9 (95% CI: 19.8–26.5) months of the supportive care group and HMA±chemo group, respectively. The median OS of allo‐HSCT was not reached. The comparison of OS curve was shown in Figure 2.

Median follow‐up was 26.1 (0.4–181.3) months. Median OS was 32.2 (95% CI: 29.3–39.6) months. Patients’ subsets were stratified according to IPSS‐R into five groups: very low (17), low (158), intermediate (166), high (162), and very high (130). As expected, the IPSS‐R risk group was strongly associated with OS (*p* < 0.001) as shown in Figure 1A. Median OS was 48.4 (95% CI: 43.6–53.7) months after censoring patients with treatment of HMA, chemotherapy, and allo‐HSCT. Patients in the five groups were very low (17), low (139), intermediate (117), high (83), and very high (63). Consistent with the results before, the IPSS‐R was markedly related to OS (Figure 1B). Four hundred and ten patients received supportive care with a median OS of 48.0 (95% CI: 43.2–53.3) months. Hundred and eighty‐eight patients received HMA±chemo with a median OS of 22.9 (95% CI: 19.8–26.5) months. The median OS of 35 patients who had allo‐HSCT was not reached. The comparisons of OS curves were shown in Figure 2.

**FIGURE 3:**
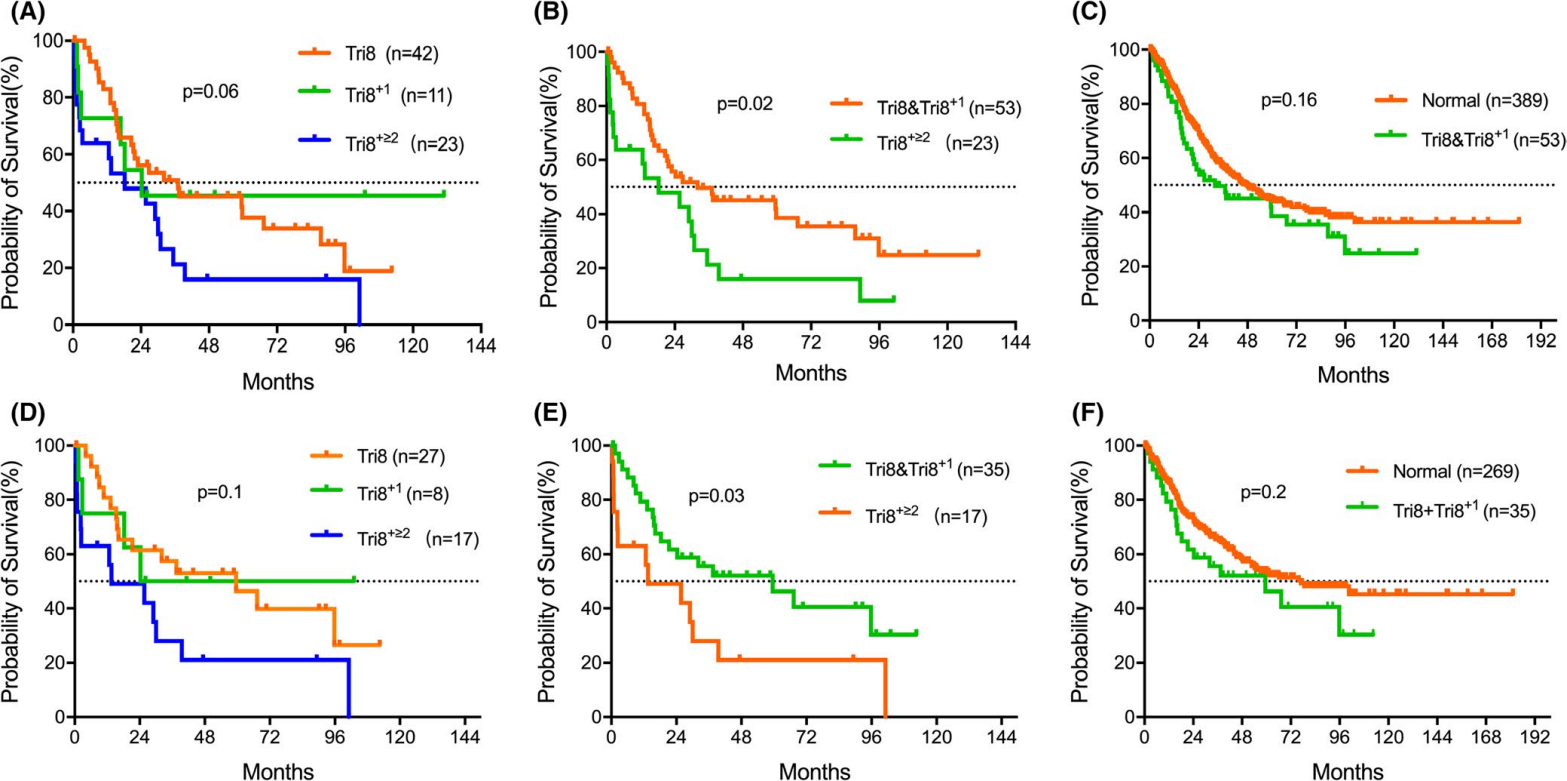
Impact of karyotype on overall survival Comparisons of survival (Kaplan–Meier curves) in all patients between tri8, tri8^+1^ and tri8^+≥2^ (A), tri8&tri8^+1^ and tri8^+≥2^ (B), normal karyotype and tri8&tri8^+1^ (C). Comparisons of survival between tri8, tri8^+1^, and tri8^+≥2^ (D), tri8&tri8^+1^ and tri8^+≥2^ (E), normal karyotype and tri8&tri8^+1^ (F) after censoring patients for treatments (HMA, chemotherapy, and allo‐HSCT).

We categorized +8 abnormality into three groups: sole +8 (tri8), +8 with one cytogenetic abnormality (tri8^+1^), and +8 with ≥2 abnormalities (tri8^+≥2^). The OS between the three groups was not significantly different (*p* = 0.06; Figure 3A). In addition, the OS between subgroup tri8 and tri8^+1^ was similar (*p* = 0.84). Then we recategorized patients into two groups: tri8&tri8^+1^ and tri8^+≥2^, the median OS were 32.1 (95% CI: 24.3–42.3) months and 18.3 (95% CI: 11.7–28.8) months respectively (*p* = 0.02; Figure 3B), whereas the OS of group tri8&tri8^+1^ showed no significant difference compared with NK group (*p* = 0.16; Figure 3C). Moreover, we compared the OS between indicated groups after censoring patients with treatment (HMA, chemotherapy, and allo‐HSCT) and discovered that the survival difference between tri8&tri8^+1^ and tri8^+≥2^ remained significant (Figure 3D‐F). Our data were in accordance with IPSS/IPSS‐R, which rank +8 as an intermediate‐risk abnormality.

**FIGURE 4:**
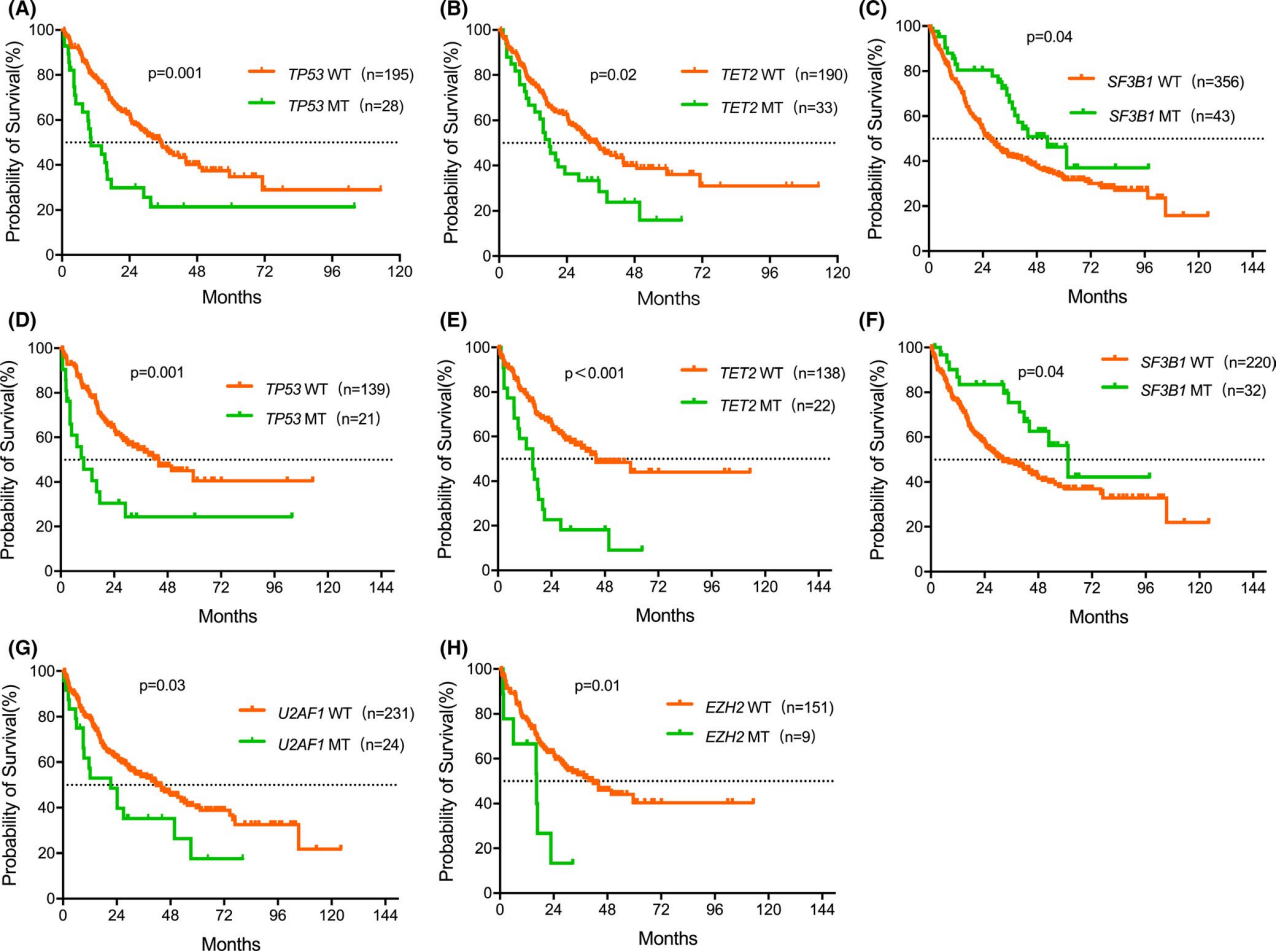
Survival curves were associated with genetic mutations Kaplan–Meier survival curves for OS of patients stratified by mutations: patients with *TP53* mutations or not (A), patients with *TET2* mutations or not (B), and patients with *SF3B1* mutation or not (C). When censoring was done for patients under treatment with HMA, chemotherapy, and allo‐HSCT, the OS of patients is significantly related to *TP53* mutations (D), *TET2* mutations (E), *SF3B1* mutations (F), *U2AF1* mutations (G), and *EZH2* mutations (H).

The study revealed that the OS of patients with mutated *TP53* or *TET2* was significantly shorter in comparison with wild type patients (*p* = 0.001 and *p* = 0.02 with *TP53* and *TET2* respectively; Figure 4A and B). Moreover, patients with mutated *SF3B1* had significantly improved OS compared with wild type patients (*p* = 0.04; Figure 4C). In addition, when censoring the patients who received treatment with HMA, chemotherapy, or allo‐HSCT, genetic mutations in *TP53*, *TET2*, *SF3B1*, *U2AF1*, *EZH2* were found to be markedly associated with OS (*p* < 0.05; Figure 4D‐H).

The univariable analysis showed that male age ≥60 years old, *TP53* mutations, *TET2* mutations, multiple genetic mutations, and marrow blasts ≥5% indicated shorter OS (*p* < 0.05; respectively), whereas *SF3B1* mutations indicated longer OS (*p* = 0.04). Multivariable analysis identified age ≥60 years old, blasts ≥5%, and *TP53* mutations as independent risk factors for worse OS, whereas *SF3B1* mutations retained an independent superior factor (Table 5). Eighty‐seven (20.5%) of 425 patients transformed to acute myeloid leukemia during follow‐up. Median LFS was not reached. Male *IDH1*/*2* mutations, *SRSF2* mutations, and blasts ≥5% showed shorter LFS in univariate analysis (*p* < 0.05). In multivariable analysis, male *IDH1*/*2* mutations and blasts ≥5% retained independent poor factors (*p* < 0.05; Table 6).

**TABLE 5:** Prognostic variables affecting survival.

Variables	Univariable analysis		Multivariable analysis	
	HR (95% CI)	*P*	HR (95% CI)	*P*
*TP53*	2.32 (1.447–3.72)	**0.001**	1.839 (1.083–3.123)	**0.024**
*EZH2*	1.818 (0.886–3.73)	0.103	1.305 (0.589–2.892)	0.512
*SF3B1*	0.618 (0.391–0.976)	**0.039**	0.496 (0.259–0.947)	**0.034**
*U2AF1*	1.391 (0.965–2.005)	0.077	0.968 (0.496–1.891)	0.924
*NRAS*	2.062 (0.961–4.426)	0.06	1.292 (0.544–3.067)	0.562
*DNMT3A*	1.248 (0.678–2.637)	0.401		
*IDH1*	1.111 (0.549–2.246)	0.769		
*IDH2*	1.35 (0.717–2.541)	0.352		
*TET2*	1.753 (1.129–2.722)	**0.01**	1.427 (0.875–2.327)	0.154
*JAK2*	1.268 (0.589–2.728)	0.544		
*CBL*	0.715 (0.264–1.938)	0.509		
*ETV6*	1.123 (0.156–8.068)	0.908		
*SRSF2*	1.251 (0.716–2.184)	0.432		
*ASXL1*	1.191 (0.670–2.117)	0.551		
*RUNX1*	1.639 (0.980–2.741)	0.06	1.208 (0.663–2.199)	0.537
−7/7q‐	0.786 (0.252–2.454)	0.678		
+8	0.927 (0.594–1.448)	0.74		
Genetic mutation counts	1.213 (1.063–1.384)	**0.004**	1.188 (0.899–1.571)	0.226
Age ≥60 years	1.983 (1.555–2.528)	**<0.001**	2.161 (1.466–3.187)	**<0.001**
Gender (female/male)	0.651 (0.507–0.836)	**<0.001**	0.809 (0.553–1.182)	0.272
Marrow blasts (%)	2.091 (1.635–2.674)	**<0.001**	1.846 (1.263–2.699)	**0.002**

The bold values here indicate *p* values which are statistically significant (*p* < 0.05).

**TABLE 6:** Prognostic variables affecting leukemia transformation.

Variables	Univariable analysis		Multivariable analysis	
	HR (95% CI)	*P*	HR (95% CI)	*P*
*TP53*	0.606 (0.188–1.958)	0.403		
*EZH2*	1.467 (0.455–4.736)	0.521		
*SF3B1*	0.414 (0.151–1.134)	0.086	0.582 (0.206–1.643)	0.307
*U2AF1*	0.936 (0.451–1.942)	0.859		
*NRAS*	0.663 (0.09–4.808)	0.684		
*DNMT3A*	1.807 (0.833–3.92)	0.135	1.101 (0.478–2.536)	0.822
*IDH1*	2.482 (1.005–6.13)	**0.049**	3.291 (1.251–8.656)	**0.016**
*IDH2*	3.827 (1.765–8.296)	**<0.001**	2.704 (1.056–6.926)	**0.038**
*TET2*	0.734 (0.289–1.858)	0.518		
*JAK2*	0.371 (0.051–2.693)	0.327		
*CBL*	0.945 (0.229–3.904)	0.937		
*ETV6*	0.001 (0‐inf)	0.997		
*SRSF2*	2.266 (1.046–4.91)	**0.038**	0.897 (0.332–2.422)	0.830
*ASXL1*	0.679 (0.211–2.191)	0.518		
*RUNX1*	0.611 (0.189–1.97)	0.409		
−7/7q‐	1.113 (0.155–7.994)	0.915		
+8	0.984 (0.454–2.132)	0.967		
Genetic mutation counts	1.044 (0.818–1.33)	0.729		
Age ≥60 years	1.016 (0.665–1.555)	0.940		
Gender (female/male)	0.439 (0.273–0.710)	**<0.001**	0.371 (0.213–0.645)	**<0.001**
Marrow blasts (%)	4.345 (2.632–7.175)	**<0.001**	3.856 (2.202–6.751)	**<0.001**

The bold values here indicate *p* values which are statistically significant (*p* < 0.05).

## 4 DISCUSSION

MDS is a highly heterogeneous group of malignancies derived from hematopoietic stem cells. The incidence rate of MDS is about 5/100,000 in population. The annual incidence rate in the elderly over 60 years old is as high as 20–50/100,000 in population and increases with age.^32^, ^33^ The median age of MDS patients in western countries is ≥70 years old,^8^, ^34^ but less than 60 years old in Asian countries.^9^, ^11^, ^35^, ^36^, ^37^ The median age of patients in our group is 57 years old, which also confirmed that the age of MDS in the Asian population was relatively young. The incidence of MDS has a gendered tendency, with more in male than in female.^6^, ^7^, ^8^, ^9^, ^10^, ^11^, ^37^, ^38^

**FIGURE 5:**
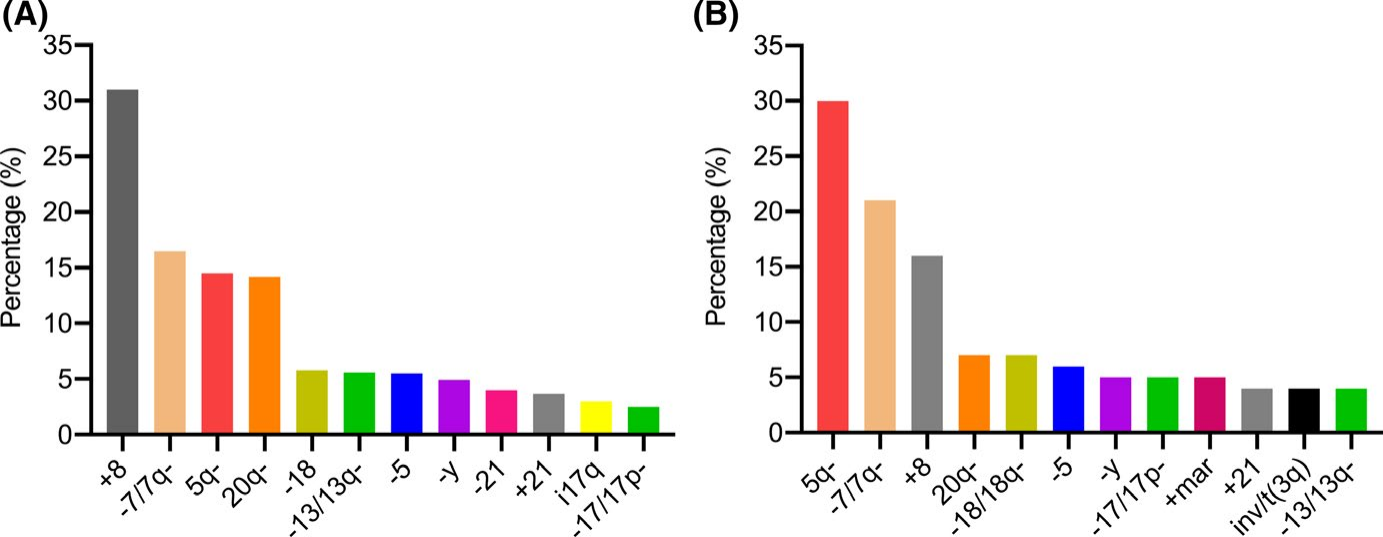
Frequency of common karyotype aberrations of MDS patients. (A) Chinese data were collected from our center and Institute of Hematology and Blood Diseases Hospital, Peking Union Medical College.^9^ (B) Caucasian cohort included 1981 primary MDS patients and 143 patients diagnosed with secondary MDS from four institutions in Austria (Hanusch Hospital, Elisabethinen Hospital, University of Vienna, and Innsbruck Medical University) and four institutions in Germany (University of Düsseldorf, University of Göttingen, University of Freiburg, and Johannes Hospital).^6^

Cytogenetic abnormalities are common in MDS (35%–51%). Our current study found that 38.6% of patients with MDS were carrying clonal cytogenetic abnormalities, which is consistent with previous studies.^6^, ^7^, ^8^ The most frequently occurring abnormality was +8, followed by −5/5q‐, −7/7q‐, 20q‐, −13/13q‐, −11/11q‐, and ‐Y. In patients from western countries, 5q‐ is the most common (30%) abnormality, whereas +8 is only identified from 11.3% to 16.0%.^6^, ^8^, ^10^ However, among Chinese MDS patients, +8 (30%‐37.8%) is the most frequent abnormal karyotype.^9^, ^11^, ^12^ In this study, +8 (31%) was the most common abnormal karyotype and more frequent than 5q‐ (20%). We compared demographics and aberrant karyotypes with Chinese and a broad group of Caucasian patients and confirmed the previous findings (Table 7). We presume the difference between Asian and western patients might be related to racial disparity (Figure 5).

**TABLE 7:** Demographics of Asian and Caucasian MDS with karyotypic aberrations.

	Our data	China^9^, ^10^, ^11^, ^33^, ^34^, ^a^	Japan^36^	Korea^35^	America^10^	Austria^32^	Spain^8^	Austria and Germany^6^
Total patients	634	2025	288	227	1363	386	968	2124
Gender								
Male, N (%)	369/58.2	1244 (61.4)	197 (68.4)	143 (63.0)	919 (67.4)	181 (46.9)	553 (57.1)	1197 (56.4)
Female, N (%)	265/41.8	781 (38.6)	91 (31.6)	84 (37.0)	444 (32.6)	205 (53.11)	415 (42.9)	927 (43.6)
Median age, years	57	48/49/57/58	69	57	66	73	70	65.7
Cytogenetic Information, N	634	1873	264	119	1363	256	968	2072
Cytogenetic Abnormalities (%)	245 (38.6)	897 (47.9)	140 (53.0)	52 (43.7)	707 (51.9)	183 (71.5)	454 (46.9)	1084 (52.3)
+8, %	31.0	31.0	12.9^b^	13.5^b^	11.3	9.8^b^	12.3	16.0
−7/7q‐, %	20	14.5	13.6	3.8^b^	7.1^b^	11.5^b^	9.5^b^	21.0
20q‐/−20, %	16.3	14.2	2.9^b^	NA	5.1	4.9^b^	2.9^c^	7.0^c^
5q‐/−5, %	20	13.3	2.9^b^	3.8^b^	10.3^b^, ^c^	32.2^b^	12.2^c^	30.0^c^

^a^ Data collected from Institute of Hematology and Blood Diseases Hospital, Chinese Academy of Medical Sciences and Peking Union Medical College, 24 hospitals in Shanghai, First Affiliated Hospital of Soochow University.

^b^ Sole Chromosome Abnormalities.

^c^ Exclusively 5q‐ in 5q‐/‐5, 20q‐ in 20q‐/‐20, and −7 in −7/7q‐.

Trisomy 8 was considered an intermediate risk factor. Conflicting data exist about the impact of trisomy 8 on OS of patients with MDS. Consistent with prior reports,^15^, ^16^, ^18^, ^39^ we found that patients with +8 had a markedly shorter OS in comparison with those who had NK (median survival 26.8 months vs. 47.5 months, *p* = 0.003). The analysis of Zoe et al. included 496 MDS patients with karyotypic abnormalities from the Victorian Cancer Registry and showed that +8 was identified in 93 (18.75%) patients and independently predicted shorter OS in a multivariate analysis (*p* = 0.024).^7^ Haase et al. analyzed 2124 MDS patients at eight institutions from Australia and Germany and found that +8 correlated with worse OS only in the patients with CK, which is consistent with our results. Median OS of +8 was 22 months and 44 months as an isolated abnormality and together with other abnormalities excluding CK, respectively.^6^

As heterogeneous prognosis exists in patients with +8, we categorized +8 abnormality into three groups (tri8, tri8^+1^, and tri8^+≥2^). The OS of the tri8^+1^ group was similar compared with that of tri8 (*p* = 0.84). The median OS of patients with tri8&tri8^+1^ was 32.1 months, which is not significantly different from those with NK (*p* = 0.16). Whereas survival was inferior in patients with tri8^+≥2^ vs tri8&tri8^+1^ patients (*p* = 0.02). This finding was similar to the phenomenon observed in MDS with 5q‐, which demonstrated that del(5q) with one additional abnormality except −7/del(7q) had the same biological characteristics as sole 5q‐, but not as 5q‐ with two or more abnormalities.^1^

Seventy to ninety percent of MDS patients displayed at least one genetic mutation surveyed according to the NGS.^20^, ^21^, ^22^, ^23^ In this study, 204 out of 425 patients (48%) had at least one mutated gene. Consistent with previous investigations, the study showed that mutated *SF3B1* was an independent predictor for improved survival. *TP53* mutations were associated with CK and poor prognosis.^28^, ^40^ Mutations in *IDH1* and *IDH2* were recognized as independent factors for leukemia transformation. We also found that *DNMT3A* mutations were more likely in patients with ‐Y, and *NRAS* mutations in 20q‐. We identified 44.9% of MDS patients with +8 had at least one mutation, but no significant association was found between +8 and distinct genetic mutations in this cohort.

There were several limitations to this study. Firstly, in this single‐center retrospective study, 427 (67.4%) patients had grown 20 metaphases under cytogenetic analysis, whereas the rest 207 (32.6%) had grown 3–19 metaphases, and FISH was undertaken as a compensatory method to identity cytogenetic abnormalities only in 74 patients. Secondly, due to technical limitation and historical background, even though 223 (52.5%) patients used NGS to detect mutations in 15 most common genes, there were also 202 (47.5%) patients who had Sanger's sequencing with a small panel of 6 genes.

Notwithstanding the limitations, our study analyzed 634 Chinese MDS patients and showed that trisomy 8 is the most common karyotypic abnormality among Chinese MDS patients. Patients with +8 showed a poor OS compared with those with NK. Sole +8 and +8 with one additional karyotypic abnormality had a similar OS with NK, whereas +8 with two or more abnormalities had a significantly shorter OS. *DNMT3A* mutations correlated with ‐Y and *NRAS* mutations correlated with 20q‐. *TP53* mutations were associated with CK and a poor OS, *SF3B1* mutations were associated with a favorable OS.^41^, ^42^, ^43^ *IDH1* and *IDH2* mutations independently indicated a shorter LFS.^44^ This study showed that cytogenetic and molecular genetic abnormalities had a significant influence on the prognosis of MDS.

## ETHICS APPROVAL

This article does not contain any studies with animals performed by any of the authors. All procedure performed in studies involving human participants were in accordance with the ethical standards of the institutional and national research committee and with the 1964 Helsinki declaration and its later amendments or comparable ethical standards.

## CONFLICTS OF INTEREST

All authors declare no conflicts of interest.

## AUTHORS’ CONTRIBUTIONS

HT and XY conceived and designed the study. LW, LJ, YL, and PL analyzed and arranged the data. WY, YR, LM, XZ, LY, GX, WX, HY, and CL provided patient samples and data. JJ guided the research with valuable comments. HT provided critical revision and suggestions.

## Data Availability

The authors confirm that the data supporting the findings of this study are available within the article.
